# Intra-host and intra-household diversity of influenza A viruses during household transmissions in the 2013 season in 2 peri-urban communities of South Africa

**DOI:** 10.1371/journal.pone.0198101

**Published:** 2018-05-24

**Authors:** Ziyaad Valley-Omar, Preetha Iyengar, Claire von Mollendorf, Stefano Tempia, Alexandra Moerdyk, Orienka Hellferscee, Neil Martinson, Meredith McMorrow, Ebrahim Variava, Katlego Masonoke, Adam L. Cohen, Cheryl Cohen, Florette K. Treurnicht

**Affiliations:** 1 Centre for Respiratory Diseases and Meningitis, National Institute for Communicable Diseases of the National Health Laboratory Service, Johannesburg, South Africa; 2 Division of Medical Virology, Department of Pathology, University of Cape Town, Cape Town, South Africa; 3 Epidemic Intelligence Service, Centers for Disease Control and Prevention, Atlanta, Georgia, United States of America; 4 Influenza Program, Centers for Disease Control and Prevention, Pretoria, South Africa; 5 Influenza Division, National Center for Immunization and Respiratory Diseases, Centers for Disease Control and Prevention, Atlanta, Georgia, United States of America; 6 School of Public Health, Faculty of Health Sciences, University of the Witwatersrand, Johannesburg, South Africa; 7 School of Pathology, Faculty of Health Sciences, University of the Witwatersrand, Johannesburg, South Africa; 8 Perinatal HIV Research Unit, Faculty of Health Sciences, University of the Witwatersrand, Johannesburg, South Africa; 9 Department of Internal Medicine, Faculty of Health Sciences, University of Witwatersrand, Johannesburg, South Africa; 10 Global Immunization Monitoring and Surveillance, Expanded Programme on Immunization, Department of Immunization, Vaccines and Biologicals, World Health Organization, Geneva, Switzerland; Icahn School of Medicine at Mount Sinai, UNITED STATES

## Abstract

Limited information is available on influenza virus sequence drift between transmission events. In countries with high HIV burdens, like South Africa, the direct and indirect effect of HIV on influenza sequence drift between transmission events may be of public health concern. To this end, we measured hemagglutinin sequence diversity between influenza transmission events using data and specimens from a study investigating household transmission dynamics of seasonal influenza viruses in 2 peri-urban communities in South Africa during the 2013 influenza season. Thirty index cases and 107 of 110 eligible household contacts were enrolled into the study, 47% (14/30) demonstrating intra-household laboratory-confirmed influenza transmission. In this study 35 partial hemagglutinin gene sequences were obtained by Sanger sequencing from 11 index cases (sampled at enrolment only) and 16 secondary cases (8 cases sampled at 1 and 8 cases sampled at 2 time-points). Viral sequence identities confirmed matched influenza transmission pairs within the 11 households with corresponding sequenced index and secondary cases. Phylogenetic analysis revealed 10 different influenza viral lineages in the 14 households. Influenza A(H1N1)pdm09 strains were shown to be genetically distinct between the 2 communities (from distinct geographic regions), which was not observed for the influenza A(H3N2) strains. Intra-host/intra-household influenza A(H3N2) sequence drift was identified in 2 households. The first was a synonymous mutation between the index case and a household contact, and the second a non-synonymous mutation between 2 serial samples taken at days 0 and 4 post enrolment from an HIV-infected secondary case. Limited inter-household sequence diversity was observed as highlighted by sharing of the same influenza strain between different households within each community. The limited intra-household sequence drift is in line with previous studies also using Sanger sequencing, corroborating the presence of strict selective bottlenecks that limit sequence variance. We were not able to directly ascertain the effect of HIV on influenza sequence drift between transmission events.

## Introduction

Within each influenza virus subtype a unique genetic background exists as a result of genetic drift [1–4]. Trends in influenza sequence drift are well documented at national and international levels but are not often explored within individual transmission events as they are rarely observed [1, 5–9]. Household transmission studies (HTS) are an ideal platform to investigate intra-host and intra-household sequence evolution using both Sanger and deep sequencing technologies [1, 7, 9, 10].

Deep sequencing has revealed the previously undetected dynamic character of influenza viruses through the identification of viral quasispecies at frequencies as low as 1–3% [8]. The ability to detect low frequency viral variants and investigate viral diversity at a more sensitive scale using deep sequencing offers an opportunity to assess viral variant permutations, and the evolutionary processes responding to selective pressures [5, 6, 8, 11, 12]. On a macro scale, these sequence permutations are not necessarily more informative than less sensitive Sanger sequencing techniques, which predominantly identify viral quasispecies present at frequencies of 20% or greater [11]. Sanger sequencing can be used to observe sequence drift and changes in the dominant viral population that exerts the most relevant effect on the host. Initial intra-host/household influenza evolution studies using Sanger sequencing have shown contrasting results demonstrating either the presence or absence of sequence evolution within households and infected individuals [1, 7, 9, 10]. These studies differed in the number of study participants as well as the number of gene segments sequenced, with sequence evolution detected predominately in studies using greater numbers of study participants and gene segments sequenced.

A recent case-ascertained household transmission study (HTS) investigated the secondary infection risk, serial interval, and associated risk factors for influenza transmission in household contacts of individuals infected with seasonal influenza in 2 peri-urban communities in South Africa [13]. The peri-urban communities from which study participants were drawn, Pietermaritzburg and Klerksdorp, consists of mostly single-family houses and informal dwellings, where 50% (n = 15) of households participating in the study reported having 2 or more people share a room for sleeping, previously identified as a risk factor for secondary influenza transmission. This study reported a secondary infection risk of 19% and a mean serial interval of 2.1 days [13].

In this study, we aimed to determine the presence of intra-host and intra-household hemagglutinin (HA) sequence domain 1 (HA1) changes in influenza A viruses derived from individuals enrolled in a South African household transmission study between May and October 2013.

## Materials and methods

### Study sampling

The study was conducted in two peri-urban sites located in Klerksdorp (Matlosana), North West Province and Pietermaritzburg (Msunduzi), KwaZulu-Natal Province, South Africa. This study was part of a larger study investigating transmission dynamics of seasonal influenza in South African households [13]. Index cases were identified and consented at primary care clinics when they presented with influenza-like-illness (ILI) (defined as cough and self-reported or measured fever (≥38°C) with onset in the 3 days prior to presentation), between May and October 2013 (South African influenza season). In addition each index case had to meet the following criteria: (i) test positive on a rapid influenza diagnostic test (RIDT) at the point of care using the Becton Dickinson (BD) Veritor™ system; and (ii) live with at least 2 household contacts who did not have symptoms suggestive of influenza at the time of enrolment [13]. Influenza positive RIDT results were confirmed by real-time reverse transcription polymerase chain reaction (rRT-PCR) at the National Institute for Communicable Diseases (NICD), Johannesburg, South Africa.

Household contacts were enrolled within 48 hours from the enrolment of the index case and were swabbed at enrolment and every 4 days thereafter over a 12-day period irrespective of the presence of symptoms to identify secondary cases. Nasopharyngeal swab samples were tested for influenza viruses at the satellite NICD laboratory, Cape Town, South Africa using the CDC Influenza Virus Real-time RT-PCR Influenza A/B typing kit, followed by H1/H3 subtyping panel (Cat#FluRUO-01) (International reagent resource, Manassas, Virginia, USA) and the BioRad iScript One-Step RT-PCR kit (BioRad, Hercules, California, USA) using a previously documented protocol [14].

The informed consent for participation in the study included optional HIV counselling and testing using a rapid HIV diagnostic test confirmed by ELISA if positive [13]. Similarly the consenting process for household contacts included optional HIV testing and contacts were enrolled irrespective of consent to HIV testing. The median age and corresponding interquartile ranges of index cases (n = 30) and enrolled secondary cases (n = 107) were 20 years (7–35) and 13 years (3–29) respectively.

The study protocol was approved by the University of the Witwatersrand and KwaZulu-Natal Human research ethics committees and by the Centers for Disease Control and Prevention (CDC, Atlanta, Georgia, USA). Written informed consent or assent was obtained from all participants or their caregivers.

### Sequencing and phylogenetic analysis

Partial hemagglutinin gene (HA1) Sanger sequencing was conducted on all available clinical samples obtained from index and secondary cases following the extraction of total nucleic acid. The globular head domain (HA1) of HA was specifically selected for sequencing as evidence has demonstrated that due to its positioning on the virion surface, it is subject to a high degree of immune pressure. This immune pressure drives the incorporation of mutations and due to the HA1 domain “flexible” scaffold structure, it is capable of accommodating mutation at a higher frequency than most other influenza gene segments [15]. The incorporation of mutations into the HA1 domain has previously been demonstrated within households with influenza A(H3N2) and A(H1N1)pdm09 infections using Sanger sequencing[1].

Roche MagNA Pure LC automated extractor (Roche Molecular systems, Mannheim, Germany) along with the corresponding Roche total nucleic acid isolation kit (Roche Diagnostics, Mannheim, Germany) was used. cDNA was synthesised utilising a universal influenza primer, uni12w with the RevertAid First Strand cDNA Synthesis Kit (Thermo Scientific, Waltham, Massachusetts, USA) according to manufacturer’s instructions, which primed synthesis of all influenza A genomic cDNA segments [16]. The influenza A HA1 gene segment was PCR-amplified using nested PCR using the World Health Organization Influenza Sequencing Primers and Protocol 45(2009). Specifically the H1F1 **/** H1R1264 (1264 bases) and H3A1F6 / H3A2R1 (1127 bases) primer sets were used for the first round of PCR and the H1F848 / HARUc (945 bases) and H3A1F3 / HARUc (863 bases) primer sets were used for the second round of PCR for A(H1N1)pdm09 and A(H3N2) respectively [17]. PCR products were directly sequenced using the ABI PRISM dye terminator cycle-sequencing kit V3.1 (Applied Biosystems, Foster City, California, USA) with the HA(H1) F and R primer set. Seventeen influenza A(H1N1)pdm09 (6 households) and 18 influenza A(H3N2) (8 households) virus sequences from the 2013 HTS were aligned using Multiple Sequence Comparison by Log Expectation (MUSCLE), with corresponding influenza subtype sequences derived from the South African Viral Watch influenza surveillance program and international reference sequences between 2011 and 2014 using default settings (GenBank accession numbers KY451418 to KY451452) [18, 19]. For the influenza A(H1N1)pdm09 alignment, 5 South African (2010 (1), 2011 (2), 2013 (1) and 2014 (1)) and 1 historical influenza A(H1N1)pdm09 reference sequences were used (A/California/07/2009). For the influenza A(H3N2) alignment, 4 South African (2012 (1), 2013 (2) and 2014 (1)) and the 2013 Southern Hemisphere A(H3N2) vaccine strain reference sequence, A/Victoria/361/2011, were used. Household phylogenies and intra-host/household nucleotide changes of influenza A(H1N1)pdm09 and A(H3N2) virus HA1 sequences were determined by the construction of maximum likelihood phylogenetic trees using RaxML (Heidelberg Institute for Theoretical Studies, Heidelberg, Germany) with the GTR-GAMMA nucleotide substitution model with branch support assessed with 1000 bootstrap replicates [20].

## Results

**Fig 1:**
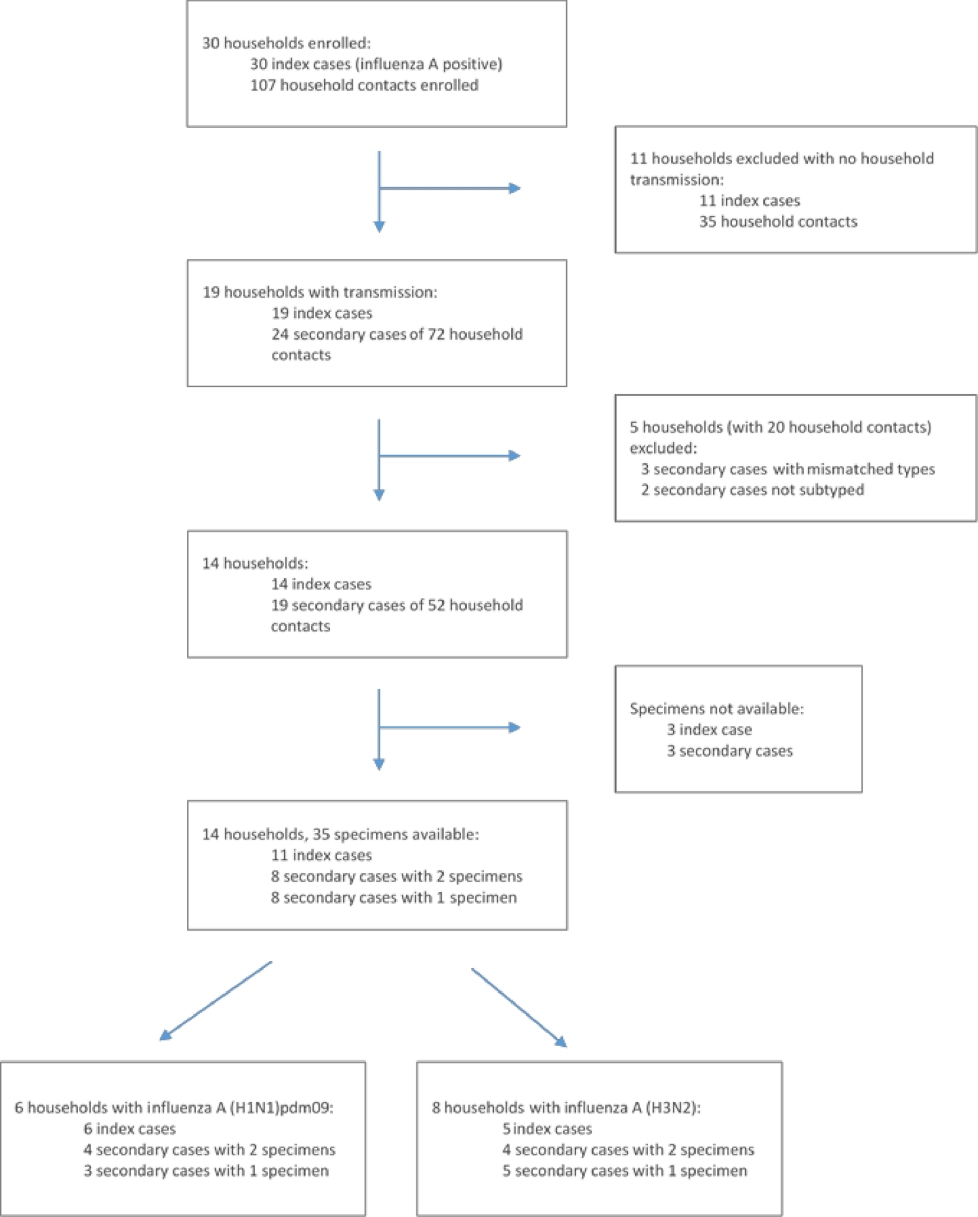
2013 Household transmission study design and sample selection [13].

Thirty index cases were enrolled, 9 (30%) of 30 tested were HIV infected, 3 of which were taking antiretrovirals, the remaining 21 (70%) were confirmed HIV negative. Within their 30 households, there were 110 eligible household contacts, of whom 107 (97%) were enrolled and 12/107 (11%) were confirmed as HIV-infected, of which 8 were taking antiretrovirals (Fig 1 and Table 1) [13]. The HIV prevalence among individuals aged 2 years and older for Kwazulu-Natal and North-West provinces where our study was conducted was 17.4% (range 15.8–19.2%) and 13.9% (range 12.0–16.1%) respectively in 2012 [21]. Three household contacts were excluded as they were not available for follow-up visits. Of the 107 enrolled household contacts, 24 (22.4%) tested influenza-positive from 19 households during the study period. Among those with available information 3% of index cases (1/29) and 5% of household contacts (5/106) reported receiving one dose of influenza vaccine in the previous 12 months, which included the current influenza season. Of our analysed sample set only the secondary case from household 101 received the influenza seasonal vaccine (Table 1). Five households were excluded from our analysis as influenza types in contacts did not match that of the index case and a further 2 secondary cases could not be subtyped due to low viral load. Therefore only14 households remained in the study of which 6 were infected with influenza A(H1N1)pdm09 and 8 were infected with influenza A(H3N2).

**Table 1:** Households with secondary transmissions of influenza A(H1N1)pdm09 (n = 6) and A(H3N2) (n = 5) investigated for drift in the hemagglutinin gene, South Africa, 2013.

**A(H1N1)pdm09**					
**Household ID**	**Community**	**Participant ID**	**HIV Status at enrolment**	**Participant Age** **(Years)**	**Influenza positive time point/s post enrolment (days)**
**001**	Klerksdorp	00 (index)	Negative	8	0
		01	Unknown	39	0, 8
		05	Negative	63	0, 4
**002**	Klerksdorp	00 (index)	**Positive**	5	0
		02	Unknown	2	0
**003**	Klerksdorp	00 (index)	Negative	6	0
		03	Unknown	26	4, 8
**004**	Klerksdorp	00 (index)	Negative	5	0
		05	Negative	2	0
**101**	Klerksdorp	00 (index)	**Positive**	48	0
		01	Unknown	53	8
**208**	Pietermaritzburg	00 (index)	Negative	33	0
		01	Unknown	29	4, 8
**A(H3N2)**					
**Household ID**	**Community**	**Participant ID**	**HIV Status**		**Time point/s post enrolment (days)**
**310**	Pietermaritzburg	00 (index)	Negative	33	0
		04	Unknown	18	0, 4
		05	Unknown	2	0, 4
**316**	Pietermaritzburg	00 (index)	**Positive**	37	0
		01	Negative	9	0
**403**	Klerksdorp	00 (index)	Unknown	20	0
		03	Negative	13	4, 8
**404**	Klerksdorp	00 (index)	Negative	16	0
		04	Negative	7	12
**408**	Klerksdorp	00 (index)	**Positive**	40	0
		04	**Positive**	3	0, 4
					

ID: Study identification number. Day 0 is the day of enrolment: within 72 hours of symptom onset for index case and within 48 hours of index case enrolment for household contacts

**Fig 2:**
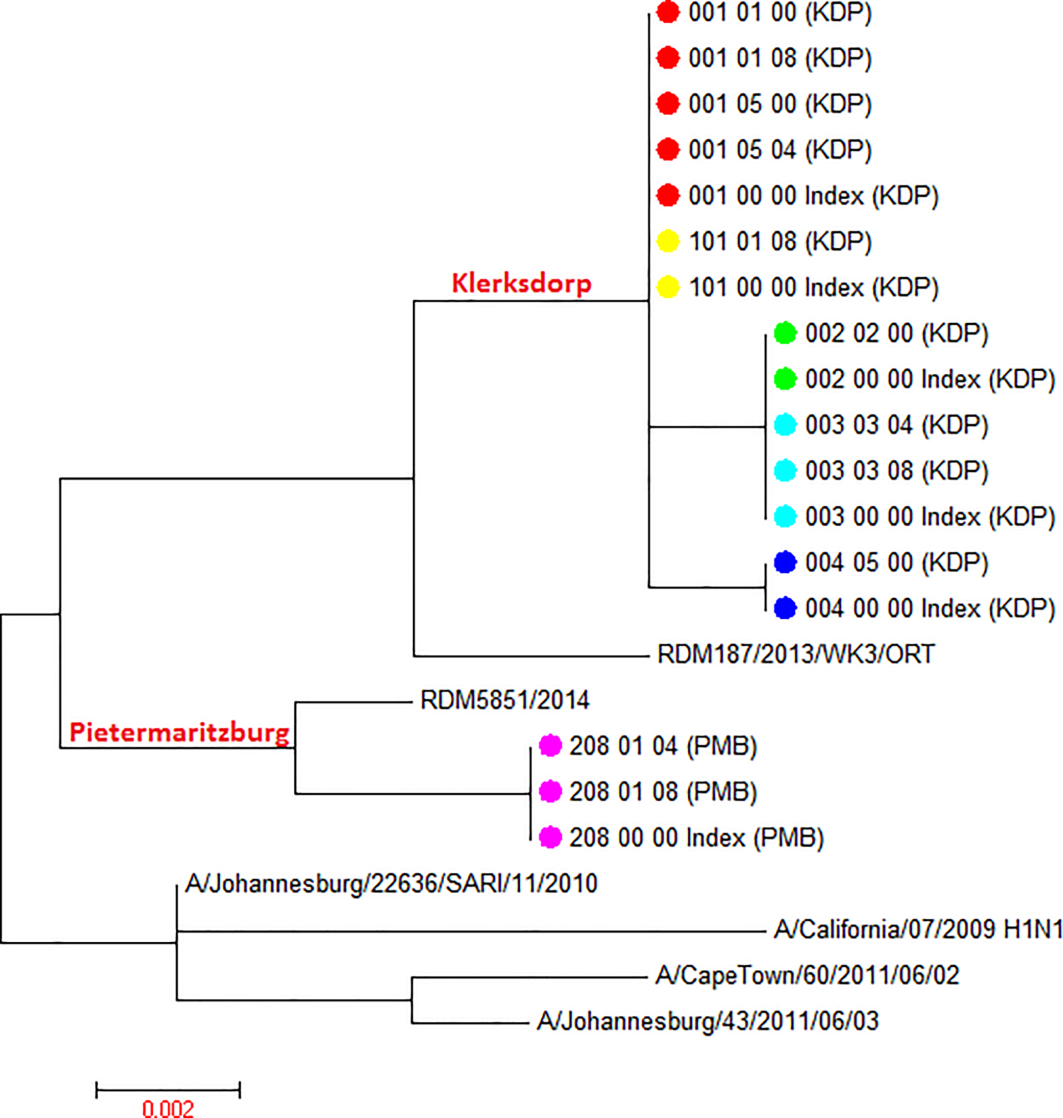
Maximum likelihood tree displaying phylogeny for influenza A(H1N1pdm09) HA1 sequences derived from household transmission study index cases and household contacts. Sequences from 4 lineages are derived from 6 households. Study sequences aligned with 5 geographic control influenza HA sequences derived from South Africa between 2010 and 2014 as well as a historical A(H1N1)pdm09 reference, A/California/07/2009. Corresponding household samples are marked with the same -colour symbols at terminal nodes; geographic separation of branches is indicated on tree highlighted in red font. Study sample sequence nomenclature: Household ID_Study participant ID_Time point post-enrollment_Sample ID_(location ID). Pietermaritzburg (PMB); Klerksdorp (KDP). The scale bar represents genetic distance between sequences (nucleotide substitutions per site).

**Fig 3:**
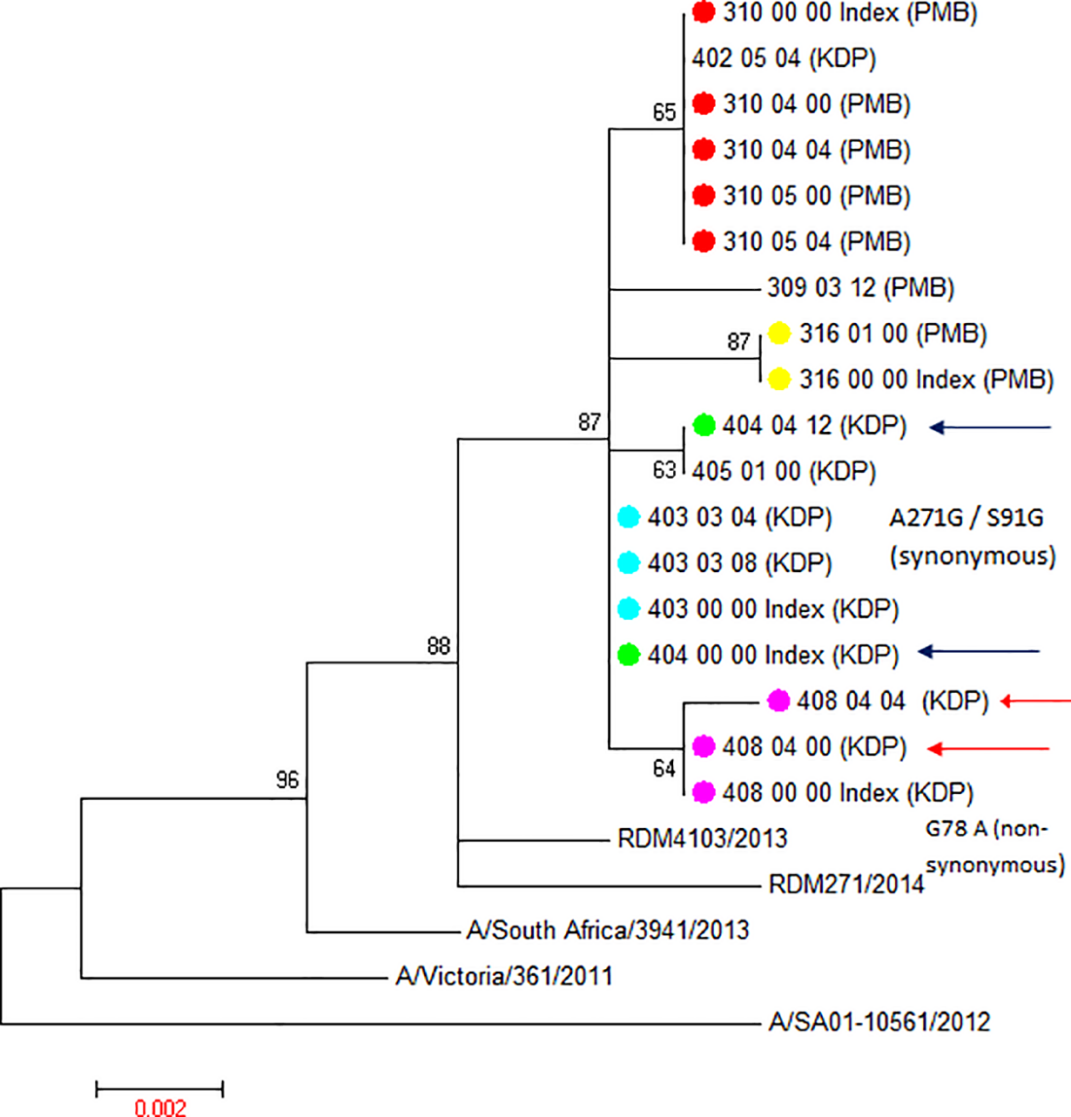
Maximum likelihood tree displaying phylogeny for influenza A(H3N2) HA1 sequences (18) derived from household transmission study index cases and household contacts. Sequences from 6 lineages are derived from 8 households. Study sequences aligned with 4 geographic control influenza HA sequences derived from South Africa between 2012 and 2014 as well as 2013 Southern Hemisphere H3N2 vaccine strain reference sequence, A/Victoria/361/2011. Corresponding household strains are marked with the same colour symbols at terminal nodes. Arrows highlight serial samples displaying sequence drift. Sequence nomenclature: Household ID_Study participant ID_Time point post-enrollment_Sample ID_(location ID). Pietermaritzburg (PMB); Klerksdorp (KDP). The scale bar represents genetic distance between sequences (nucleotide substitutions per site).

A total of 35 samples collected from 11 index (1 time-point only, n = 11) and 16 secondary cases (1 time-point n = 8, 2 time-points n = 8) from the 14 households were available for sequencing (Fig 1). Table 1 summarizes the demographics for 11/14 households where at least one secondary contact is available for each index case. The samples from 3 index cases could not be retrieved and are excluded in Table 1. (Households 309, 402 and 405). Six households had influenza A(H1N1)pdm09 transmissions and 5 households influenza A(H3N2) transmissions. Across subtypes, 10 unique viral variants were observed in the 11 households sequenced (Figs 2 and 3).

The influenza A(H1N1)pdm09 strains from 6 households separated into 2 distinct branches, each specific for the 2 geographic locations from which the households were enrolled (Fig 2). Influenza A(H1N1)pdm09 viruses from 5 Klerksdorp households showed 3 distinct sub-lineages defined by 16 and 17 nucleotide changes (1 or 2 amino acid changes) when compared to the A/California/07/2009 reference sequence. The single household (208) from Pietermaritzburg distinctly differed from the Klerksdorp household clusters displaying 16 nucleotide changes (2 amino acid changes) compared to the A/California/07/2009 reference sequence. In contrast to the genetic differences observed in influenza A(H1N1)pdm09 strains identified between household clusters, the tight clustering of intra-household influenza A(H1N1)pdm09 sequences indicates that there were no nucleotide changes between the index and secondary cases for the HA1 domain of the hemagglutinin gene. Similarly no nucleotide changes were observed between serial samples from secondary cases.

In contrast to our influenza A(H1N1)pdm09 findings, influenza A(H3N2) viruses transmitted in 8 households (5 in Klerksdorp and 3 in Pietermaritzburg) separated into 6 distinct lineages but displayed no distinct geographic identity (Fig 3). Instead, the 6 lineages were interspersed between each other on the maximum likelihood tree and were defined by 12 to 15 nucleotide changes (6 to 7 amino acid changes) when compared to the A/Victoria/361/2011 Southern Hemisphere 2013 vaccine strain sequence (Fig 3). In the majority of household clusters we found a conserved sequence identity (no nucleotide changes) between individuals in the household (Fig 3). However in two households (404 and 408) nucleotide sequence changes were detected (Fig 3). For household 404, a nucleotide change (G30A transition) was evident between the index case (404_00_00) and the secondary case (404_04_12) (both HIV negative) in a serial sample obtained 12 days after enrolment, which resulted in a synonymous mutation (Fig 3). In household 408 sequence drift was detected between 2 serial samples taken at days 0 and 4 post enrolment in a secondary case. The secondary case day 0 sample sequence was identical to the index case sequence and the day 4 sample contained both sequence variants (not shown). This A271G transition resulted in an S91G amino acid substitution (polar to neutral amino acid). Both the index and secondary case in this household transmission pair were HIV infected and was the only HIV-infected concordant transmission pair in our study. These individuals were not receiving antiretroviral therapy and their HIV viral loads at the time of sampling are not known.

## Discussion

We assessed the intra-host and intra-household variability of influenza A subtypes in peri-urban South African households using Sanger sequencing. The prevalence of influenza virus types/subtypes identified in communities sampled, which showed dominance of A(H1N1)pdm09, was reflective of the influenza virus type/subtype distribution in South Africa during the study period [22, 23]. In the available sample set, while influenza A(H1N1)pdm09 strains did not display any intra-household nucleotide changes in the regions sequenced, a greater level of sequence diversity existed within the communities sequenced. Influenza A(H1N1)pdm09 strains appeared to exhibit a geographic identity segregating according to the communities from which the viral strains were derived, which may be an artefact of limited population sampling. We demonstrated limited nucleotide changes in 2 influenza A(H3N2)-infected households within the hemagglutinin HA1 domain. It is notable that the secondary case that demonstrated a non-synonymous, S91G HA mutation (household 408) between serial samples was the only secondary case known to be HIV infected. Studies have suggested that this specific amino acid (S91) forms part of a conserved set of amino acid residues that can serve as a neutralizing antibody binding domain. S91 specifically serves as one of several hydrogen binding sites for neutralizing antibody binding, where the S91G mutation may act by impairing the antigen-antibody interaction [24]. The HIV viral load for this individual is unfortunately unknown. It should also be noted that the 12 day period between influenza sequences derived from the index and household contact in household 404 may suggest a community-acquired infection in the secondary case and not necessarily be a result of sequence drift between successive transmission events within the household. In contrast to previous HTSs, not all households in this study could be distinguished by unique viral phylogenies as some appeared to share identical influenza strains. This may suggest limited influenza sequence variability within the communities or inter-household transmission, which may be clarified by understanding the social dynamics within these communities. The sequencing depth limitations of Sanger sequencing may have prevented us from attaining necessary sequencing depth to distinguish households with similar strains. Similarly, the lack of sequencing depth has prevented us from determining the statistical significance of intra-household and intra-host sequence identity. The use of full genome deep sequencing technologies in future studies could therefore be instrumental to detect unique ratios of viral quasispecies that exist within households or between transmission pairs with more confidence by providing a sequencing depth greater than the 20% threshold of Sanger sequencing [8].

The variability that we noted in the sequenced HA1 segments appears low, but is in agreement with genome-wide Sanger sequencing based influenza A evolution analyses conducted within 11 households (31 samples, 23 individuals) from Hong Kong which also demonstrated 1–3 nucleotide differences within households [10]. Furthermore, due to the small sample size and limited sequencing depth, no conclusions could be drawn about the effect of HIV infection on influenza sequence drift. While studies have identified HIV infection as a risk factor contributing to prolonged shedding, hospitalisation and death of influenza-infected individuals, none have documented the effect of HIV on influenza sequence drift [25]. However, culture of influenza viruses in cell lines, an immunologically non-selective system, has been shown to facilitate a rapid increase in viral diversity through an increase in mutations, which could be considered a proxy for influenza evolution in HIV-infected individuals [12]. In a country like South Africa, which has a high HIV burden and a low annual influenza vaccine coverage of approximately 2% of the population, determining the direct or indirect effects that HIV may have on influenza virus diversity is of significant public health concern [26]. Limited immunity, resulting from HIV infection and limited influenza vaccine uptake may facilitate selection to increase genetic diversity, which may facilitate the virus’s ability to escape from vaccine and drug treatment pressures. This motivates the need to continue studies of this nature with sufficient numbers of HIV-positive individuals and using the advantages of deep sequencing methodologies [12].

Results from our relatively small sample corroborate findings from prior HTSs that have shown limited sequence changes between direct transmission events [1, 10]. However, Sanger sequencing may lack the depth in sensitivity to accurately account for rare variants, and the mutations identified may therefore only represent a brief snapshot of the adaptive evolutionary process that leads to the selection of replication fit/active virus transmission as represented by high frequency variants. In contrast, deep sequencing-based studies have tended to corroborate one another showing a greater frequency of mutations between transmission events, possibly reflecting a more accurate picture of influenza evolutionary patterns [5, 6, 8, 12]. Sanger sequencing may still have utility in these types of studies as the vacillation of results could indicate that sequence changes during individual transmission events may not exceed the 20% detection threshold and are unlikely to cause a shift in quasispecies ratios [11]. However, this study does corroborate observations made using deep sequencing that have shown a strict selective bottleneck with well adapted viral variants within the individual or household [12]. The ubiquity of specific viral variants between different households within communities may suggest that they are optimally adapted.

We aimed to determine the presence of intra-host and intra-household hemagglutinin gene sequence changes from influenza viruses from individuals enrolled in a HTS. While the study was underpowered to evaluate the role of HIV-induced immunosuppression in influenza virus sequence drift we did demonstrate limited intra-host and intra-household influenza sequence drift within at least 1 household. The study also did not investigate social mixing patterns in the community which may explain the 12 day serial interval between index and secondary case in household 404 as well as the discrepant strains in index and secondary cases for 3 households. To enable a more accurate evaluation of influenza diversity, adaptation and mutation under circumstances such as concurrent HIV-infection, deep sequencing on a larger number of influenza viruses derived from HIV–infected and HIV-uninfected individuals is recommended.
